# Increase in cesarean sections in Brazil – a call to reflection

**DOI:** 10.1055/s-0043-1768454

**Published:** 2023-04-27

**Authors:** Antonio Braga, Sue Yazaki Sun, Alberto Carlos Moreno Zaconeta, Alberto Trapani Junior, Adriana Gomes Luz, Gabriel Osanan, Geraldo Duarte, José Geraldo Lopes Ramos, Maria Celeste Osório Wender, Roseli Mieko Yamamoto Nomura, Rossana Pulcineli Vieira Francisco, Vera Therezinha Medeiros Borges, Rosiane Mattar

**Affiliations:** 1 Department of Obstetrics and Gynecology, Rio de Janeiro Federal University, Rio de Janeiro, RJ, Brazil; 2 Department of Maternal Child, Fluminense Federal University, Niterói, RJ, Brazil; 3 Vassouras University, Vassouras, RJ, Brazil; 4 Department of Obstetrics, Escola Paulista de Medicina, Universidade Federal de São Paulo, São Paulo, SP, Brazil; 5 University of Brasilia, Brasília, DF, Brazil; 6 Departamento de Ginecologia e Obstetrícia, Federal University of Santa Catarina, Florianópolis, SC, Brazil; 7 Department of Obstetrics and Gynecology, School of Medical Sciences, State University of Campinas, Campinas, SP, Brazil; 8 Department of Obstetrics and Gynecology, Federal University of Minas Gerais, Belo Horizonte, MG, Brazil; 9 Department of Gynecology and Obstetrics, Ribeirão Preto Medical School, University of São Paulo, Ribeirão Preto, SP, Brazil; 10 Department of Gynecology and Obstetrics, Faculty of Medicine, Universidade Federal do Rio Grande do Sul, Porto Alegre, RS, Brazil; 11 Departamento de Obstetrícia e Ginecologia, Faculdade de Medicina, Universidade de São Paulo, São Paulo, SP, Brazil; 12 Department of Gynecology and Obstetrics, Botucatu Medical School, São Paulo State University, Botucatu, SP, Brazil

Cesarean rates have increased progressively over the decades in all countries, and a high figure of 56% was reached in Brazil, second only to the Dominican Republic (59%) and well above the average of developing countries. 1 This scenario in our country motivated government and private sector initiatives, among which the *Projeto Parto Adequado* (“Adequate Childbirth Project”), with a view to reducing cesarean sections. 2 The set of these actions allowed for a stabilization and even a slight decrease in cesarean section rates according to data from the Information System on Live Births (Portuguese acronym: SINASC) of the Brazilian Ministry of Health. However, preliminary data from SINASC for 2022 pointed to a further increase in cesarean sections in Brazil (cesarean section rates: 2016: 55.4%; 2017: 55.7%; 2018: 55.9%; 2019: 56.3%; 2020: 57.2%; 2021: 57%) 3 and motivated this reflection made by Brazilian obstetric schools.

Undoubtedly, the COVID-19 pandemic has profoundly affected healthcare in Brazil, accelerating trends and highlighting weaknesses. In the obstetric scenario, the country already showed signs of an increase in cesarean rates from 2017 onwards, and the pandemic accentuated this process. Although the healthcare network was reorganized to maintain antenatal care during the pandemic, this was limited in practice, especially for patients at obstetric risk. As a result, pregnant women arrived at maternity hospitals with obstetric complications in more severe stages and the indication for cesarean sections to alleviate an unfavorable maternal-perinatal outcome. In addition, especially at the beginning of the pandemic, although there was no explicit guidance, many cesarean sections were performed under the mistaken belief that this would bring better maternal outcomes. With experience in the management of COVID-19, it was observed that whenever plausible, the resolution should be postponed until clinical stabilization of the pregnant woman. However, in cases with a precise indication of resolution of the pregnancy because of severe conditions, the possible mode of delivery was mostly the cesarean section. 4

The fact that delivery care was challenging during the COVID-19 pandemic may also have contributed to the increase in cesarean sections. Factors such as restrictions on the presence of companion during hospitalization for childbirth in many services; lack of guidance to pregnant women for labor as participation in antenatal classes was lower because of social distancing 5 ; fear of hospitalization, perceived as a source of transmission of the disease; and increased anxiety, sadness and fear with intense psychological distress caused by the pandemic and enhanced by the disruption of the support network for pregnant women 6 may have contributed to the increase in cesarean rates in this period.

However, the COVID-19 pandemic also highlighted the fragility of the maternal and child care network in Brazil. The lack of synchronized and adequate management in the different maternity hospitals around the country and a fragile system of hierarchy for referring cases of high obstetric risk demonstrate the complexity of this problem. The continental size of our country, the heterogeneity of health services, the absence of a complete medical team each day of the week in small towns, as well as the existence of settings without implemented assistance care sometimes determine the performance of electively scheduled cesarean sections on days when the complete team is available for fear of obstetric emergency situations during labor.

Of course, it would be naive to credit the high cesarean rates in Brazil only to the COVID-19 pandemic. There are other well-known determinants that help compose this scenario, such as the lack of a culture of multidisciplinary teamwork in childbirth care; the scarce supply of pharmacological analgesia; imprecise indications for early delivery due to suspected impairment of fetal vitality and the underfunding of obstetric care, both at the institutional level and at the level of professionals who accompany deliveries. The high number of cesarean sections also ends up feeding back into this cycle, when women with previous cesarean sections are very often subjected to repeat cesarean sections because of the obstetric fear of rare cases of uterine rupture aggravated by the unavailability of prostaglandin E _2_ in the national market. Avoiding the first cesarean is strategic to break this cycle. To this end, the systematic second opinion for the indication of cesarean section and the analysis of the Robson classification for the study of cesarean sections are strategies that can avoid cesarean sections not considered as the best clinical indication.

The cesarean culture also influences the legal world and is reflected in the obstetric team's fear of being sued for lawsuits in cases of malpractice, which certainly contributes to a more “early” or unnecessary indication of cesarean section. A survey by the American College of Obstetricians and Gynecologists (ACOG) in 2015 showed that 73.6% of North American gynecologists and obstetricians suffered at least one malpractice lawsuit (62% in obstetrics and 39% in gynecology). 7 In the judicial sphere, it should be noted that the legislation regulating the performance of cesarean sections at the pregnant woman's request and the right to labor analgesia, under the aegis of autonomy, even without medical indication, may also have influenced the increase in cesarean sections. However, given the difficulty in offering analgesia, especially pharmacological, in Brazilian maternity hospitals, many women end up requesting a cesarean section during labor, which contributes to the increase in the number of cesarean sections without medical indication. It is further presumed that Federal Law Number 14.443/2022, 8 which updates the legislation on Family Planning in Brazil, effective as of March 2023, and which allows tubal ligation at the time of delivery, further increases cesarean rates. Finally, and although the Brazilian Federal Council of Medicine recognizes women's right to request a cesarean section 9 from 39 completed weeks of pregnancy, guaranteeing the autonomy of the physician and the patient and the safety of the mother-fetus dyad, it is essential to guarantee that all pregnant women can be assured of a safe delivery. Otherwise, the woman's autonomy may be weakened by the lack of equity and the request for a cesarean section will simply reflect the lack of option for a respectful and pain-free delivery.

Although the cesarean section is a life-saving surgery, representing a great advance in obstetric practice and in the integral protection of the mother-fetus dyad, its reckless performance is associated with relevant immediate and future risks. Among the immediate maternal risks of a cesarean section, are the increase in intrapartum bleeding and postpartum hemorrhage, the increased risk of maternal infection/sepsis, thromboembolic conditions and injuries to pelvic organs, especially in emergency surgery. With regard to immediate fetal risks, iatrogenic prematurity (due to early term birth) and increased rates of transient tachypnea in newborns stand out. In addition, birth trauma can occur during a cesarean section. 10 This surgery can still cause future complications such as reduced fertility, abnormal uterine bleeding and chronic pelvic pain, 10 in addition to greater risks of pregnancy in a cesarean section scar, uterine rupture and placenta accreta. These obstetric complications are responsible for severe and potentially lethal maternal hemorrhagic conditions, and are associated with significant maternal morbidity and mortality. 10 11 12 The possible future risks of fetuses born by cesarean section include alterations in the intestinal microbiome, as well as higher rates of immunological dysfunctions, metabolic disorders (such as obesity and asthma) and cognitive disorders (such as hyperactivity). 10 Considering such high cesarean rates and the immediate and future risks determined by this surgery in women and fetuses, there is need for a reflection that results in strategies to reduce unnecessary cesarean sections in our country.

The initial strategy must include antenatal actions. Health literacy will allow women to have an active role and make more appropriate informed decisions about childbirth. 13 The formation of groups of pregnant women to discuss the types of childbirth, physiology and stages of normal childbirth; encouraging the presence of a companion during antenatal care, so that they receive information and help the pregnant woman, transmitting her security; encouraging pregnant women and companions to visit the reference maternity hospital in order to provide greater security at the time of delivery; agreeing on a birth plan during antenatal care; and guidance on non-pharmacological pain control methods are measures that should be encouraged to reduce cesarean section rates.

Listening to women's expectations for their childbirth is essential for encouragement in this route of birth. In 2018, the World Health Organization (WHO) published 14 a summary of these aspirations: care provided by a sensitive, attentive, kind and respectful team; presence of a companion (having a person she chooses by her side will bring emotional security and comfort); accurate birth interventions; autonomy (being informed and participating in decisions); labor analgesia (non-pharmacological and also pharmacological whenever requested); delivery outcome with healthy mother and newborn.

Feeling pain during labor is one of women's biggest fears. Brazil faces an enormous shortage of pharmacological analgesia for childbirth. This has been identified as one of the factors that most influence the choice for a cesarean section. Few hospitals provide anesthesiologists 24 hours a day to offer labor analgesia if requested by the pregnant woman. If we want to guarantee women a pain-free delivery and thus reduce cesarean rates, access to and availability of analgesia for all women who request it is one of the main challenges to be faced. Discussing new models of anesthetic care during labor, as in countries with a wide supply of labor analgesia, such as the United States and France, may be opportune.

The need to organize the maternal and child healthcare network is urgent. Health equipment should be restructured in a rational way, reducing the number of maternity hospitals in cities with a low population rate, whose residents can be attended in regional hospitals. This restructuring will provide greater security for the mother-fetus dyad, ensuring professional structure and permanent material resources, linked to the primary objective of improving childbirth care, reserving cesarean section for the best indicated cases. In addition, these reference centers for childbirth would have an appropriate space for parturition, with LDRP models (space for labor, delivery, recovery and postpartum room), where the parturient woman and her companion would remain in an embracing environment with privacy and dignity, linked to an obstetric center that guarantees safety and prompt intervention whenever necessary. Certainly, the presence of a multidisciplinary delivery team is beneficial and associated with a reduction in cesarean rates.

Although the WHO is focused on ensuring that cesarean sections are performed whenever necessary rather than seeking to achieve a specific cesarean rate, 15 it is undeniable that Brazil performs more cesarean sections than women want or need. In this editorial, we analyzed some factors that may be associated with high cesarean rates in Brazil. The joint work of health authorities, medical societies, universities, managers and the multidisciplinary team in teaching, embracement and adequate, safe and respectful care is essential for cesarean rates to truly decrease and not rise again.
